# Changes in the periodontal and tomographic parameters of 36 anterior maxillary teeth one year after periapical surgery with submarginal incision

**DOI:** 10.4317/medoral.27157

**Published:** 2025-08-16

**Authors:** Araceli Boronat-López, Juan Cervera-Ballester, Juan Carlos Bernabeu-Mira, Miguel Peñarrocha-Diago, David Peñarrocha-Oltra

**Affiliations:** 1 Master of Oral Surgery and Implantology, Medical and Dental School, University of Valencia, Spain; 2 Full Professor, Department of Stomatology, Medical and Dental School, University of Valencia, Spain; 3 Professor and Chairman of Oral Surgery and Implantology, Medical and Dental School, University of Valencia, Spain. IDIBELL (Bellvitge Biomedical Research Institute), Barcelona, Spain

## Abstract

**Background:**

A study was made of the clinical periodontal changes and buccal cortical bone modifications using cone-beam computed tomography (CBCT) in anterior maxillary teeth with chronic apical periodontitis one year after periapical surgery with submarginal incision.

**Material and Methods:**

A prospective case series analysis was made of anterior teeth subjected to apical surgery and submarginal incision with a follow-up period of 12 months. Clinical periodontal parameters were recorded, along with tomographic measurements of the buccal cortical bone and volume of the lesion (in mm3) before and one year after surgery. Success was assessed based on the clinical and tomographic data.

**Results:**

Thirty-six anterior maxillary teeth from 36 patients with a mean age 43.1 years were enrolled in the study. One year after surgery, mean gingival recession was found to be 0.19 mm with a clinical attachment loss of 0.28 mm. Marginal bone loss was 0.25 mm. The thickness of the buccal cortical bone decreased at all three measurement points, with the greatest decrease being observed at 3 mm from the bone crest (0.58 mm). The distance from the apex to the buccal cortical bone (depth of the apex) decreased 0.59 mm at one year. The clinical parameters (clinical attachment level and probing depth) were not correlated with the tomographic measurements (cementoenamel junction-bone crest distance). The mean lesion volume was 457 mm3 at baseline versus 28.4 mm3 one year after surgery, representing a decrease of 93.8% in 12 months. The success rate at one year postsurgery was 94.4%.

**Conclusions:**

One year after apical surgery of anterior maxillary teeth with submarginal incision, only minimal clinical periodontal and tomographic changes are observed, with no clinical relevance. The mean lesion volume decreased 93.8%, and the success rate was 94.4%.

** Key words:**Apical surgery, submarginal incision, buccal bone, buccal bone thickness, periodontal parameters, healing outcome, CBCT.

## Introduction

Periapical surgery involves the raising of a soft tissue flap to access the surgical field, and the design of the flap may result in clinical periodontal changes and influence the buccal cortical bone (1). Adequate soft tissue management is essential in order to avoid damage and adverse outcomes in terms of wound healing that may influence the aesthetic success of the treatment (2,3). The type of incision can have a direct impact, and different flaps for the surgical approach have been proposed in order to minimize these changes, ranging from the traditional intrasulcular incision that mobilizes the dental papilla and all the gingival tissue in the flap; incision of the base of the papilla to preserve the interproximal papilla and periodontal tissues; to the current submarginal incision technique (3,4).

The literature describes and compares the different types of flap, with sulcular flaps and incisions of the base of the papilla being the most widely studied to date (3-7). Taschieri *et al*. (5) compared incision of the base of the papilla versus sulcular incision to evaluate changes in the height of the papilla and in gingival margin. The authors found no differences in the change in gingival margin (recession) between the two groups. Velvart *et al*. (6) compared the same incisions and found incision of the base of the papilla to result in faster and more predicTable healing than sulcular incision. In turn, Kreisler *et al*. (1) compared sulcular and submarginal incisions, with neither being seen to produce changes, though sulcular incision was associated with slight gingival recession. Von Arx *et al*. (7) evaluated the periodontal changes after apical surgery using three types of incision (intrasulcular, base of the papilla and submarginal), with significant differences being observed in terms of gingival margin and clinical attachment; specifically, submarginal incision was associated with significantly less gingival recession. On the other hand, the meta-analysis conducted by Castro-Calderón *et al*. (8) revealed no significant differences in the clinical periodontal parameters according to the type of incision used, though incision of the base of the papilla was identified as the best option in order to reduce gingival recession, followed by submarginal incision.

In the anterior maxilla, von Arx *et al*. (3) recommended avoiding flaps that involve elevation of the papilla, such as sulcular incisions, as far as possible, since they pose a greater risk of midbuccal gingival recession. Thus, given the current aesthetic demands, we conducted a study to evaluate the clinical periodontal, tomographic and aesthetic changes in the upper maxillary teeth one year after periapical surgery involving submarginal incision.

## Material and Methods

- Study design

The present prospective study was carried out at the Department of Oral Surgery (Medical and Dental School, University of Valencia, Spain) in the period between January 2020 and December 2022. The study was approved by the Ethics Committee of the University of Valencia (Ref.: 1224932), and was carried out in abidance with the principles of the Declaration of Helsinki (9). Written informed consent was obtained from all the patients, who were free to leave the study at any time. The manuscript was prepared in line with the STROBE statement (10).

- Patient selection

The study included healthy individuals (without serious systemic or functional disorders) with anterior maxillary teeth (central and lateral incisors, and canines) subjected to endodontic treatment and presenting chronic periapical lesions affecting a single tooth. All patients received an explanation of the surgical procedure, and a complete medical history was recorded, with the collection of clinical and cone-beam computed tomography (CBCT) data, before and 12 months after surgery. The exclusion criteria were: teeth lacking tomographic control or with poor quality imaging; teeth with an endoperiodontal lesion or probing depth of over 4 mm; and teeth in which the bone cavity was filled with graft or bone replacement material.

- Surgical technique

**Figure 1:**
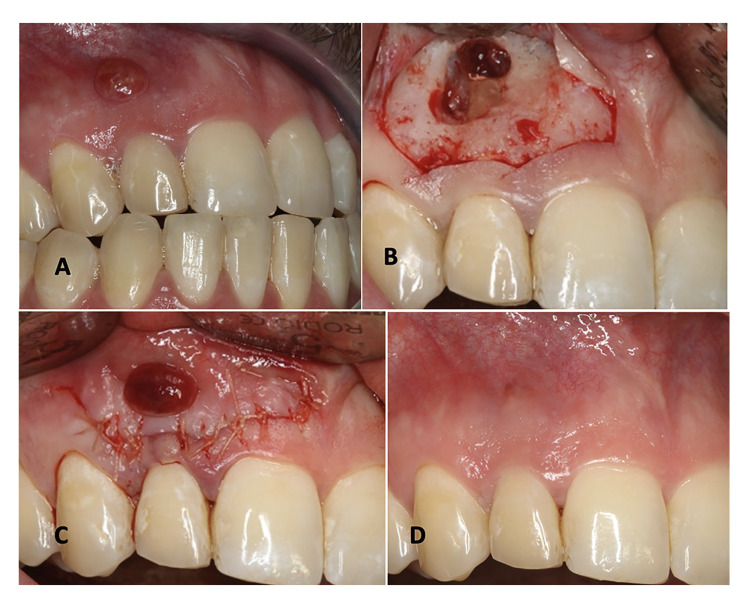
Preoperative clinical view (A), view at the time of surgery (B), view on completing suturing (C) and control 12 months after surgery (D).

Preoperative antibiotic prophylaxis was administered with 2 g amoxicillin or 600 mg clindamycin one hour before surgery. The same surgeon performed all the operations. All surgeries were carried out under infiltrative local anesthesia with 4% articaine and 1:100,000 epinephrine (Inibsa®, Lliça de Vall, Barcelona, Spain). A photograph was obtained before surgery (Fig. 1). A submarginal incision was performed (i.e., a festooned incision following the gingival contour at 2-3 mm from the attached gingiva, with 1-2 releasing incisions). Elevation of the flap was made from the releasing incision towards the cervical area (Fig. 1). Following the submarginal incision, a full thickness mucoperiosteal flap was raised and ostectomy was performed to access the root apexes and apical lesions. The affected root was resected approximately 3 mm from the apex with minimal or no bevel, and the pathological tissue was curetted out. After hemostasis, root-end cavities were prepared with ultrasonic retrotips (Piezomed, W&H Dentalwerk, Bürmoos GmbH, Bürmoos, Salzburg, Austria) to a 3 mm depth and filled with mineral trioxide aggregate (MTA) (ProRoot; Dentsply Tulsa Dental, Tulsa, OK, USA). An endoscope (Karl Storz-Endoskope, Tuttlingen, Germany) was always used for the inspection of root-end resection, cavity preparation and retrograde filling. After cleaning the bony crypt, the flap was closed and sutured with 5/0 suture material (Supramid®, B. Braun, Rubí, Barcelona, Spain), with correct adaptation and approximation of the flap without applying tension (Fig. 1).

All patients were prescribed 0.12% chlorhexidine rinses twice daily for 10 days and 600 mg ibuprofen as needed. Sutures were removed 7 days after surgery. The patients underwent tomographic and photographic control after 12 months (Fig. 1).

- Study parameters

Clinical assessment

**Figure 2:**
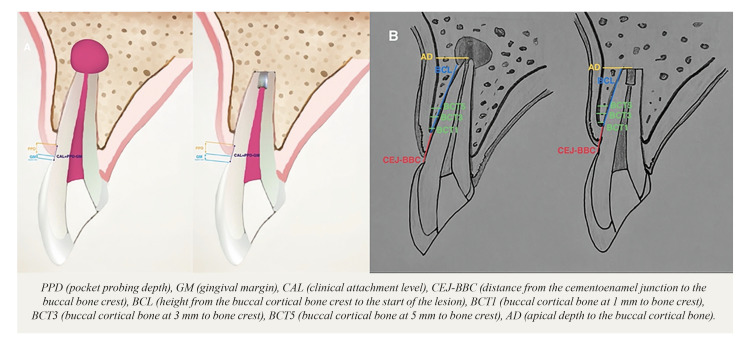
Periodontal parameters and CBCT measurements. 2A Schematic representation of the periodontal parameters at the midbuccal site preoperatively and at one year of follow-up. 2B Schematic representation of the tomographic measurements preoperatively and at one year of follow-up.

Preoperatively and 12 months year after surgery, the clinical examination included the assessment of symptoms and signs (pain, swelling and oral fistulas), together with different periodontal parameters (Fig. 2). Pain intensity was scored as: 0 = none, 1 = mild, 2 = moderate and 3 = severe.

1. Pocket probing depth (PPD) was measured using a periodontal probe (Colorvue Tip; Hu-Friedy, Leimen; Germany) to the nearest 0.5 mm at four sites: mesiobuccal, midbuccal, distobuccal and midpalatal.

2. Recession of the gingival margin (GM) was measured using the same probe, recording the distance from the GM to the cementoenamel junction (CEJ) or to the restoration margin (RM) to the nearest 0.5 mm at midbuccal aspect (with negative values for sites with exposed root surface).

3. Bleeding on probing (BOP) was scored according to Mombelli *et al*. (1987): 0 = no bleeding, 1 = isolated bleeding spots, 2 = confluent blood line, 3 = profuse bleeding.

4. Clinical attachment level (CAL) was calculated as PPD at midbuccal level minus the GM value (CAL = PPD MD - GM). As mentioned above, GM had negative values ​​in the presence of exposed root surface.

5. Fenestration was assessed on the day of surgery, on examining the buccal cortical bone, and was scored as 0 = no fenestration or 1 = presence of fenestration.

6. The aesthetic change of the tooth was recorded 12 months after surgery, taking into account midbuccal gingival recession of the tooth and the presence or not of scarring due to suturing: 0 = no aesthetic change (recession < 1 mm and no scarring) or 1 = aesthetic change (recession > 1 mm and/or presence of scarring).

Tomographic assessment

The width and height of the buccal bone plate was assessed from the sagittal section because these are anterior teeth. CBCT images were obtained with a 3D Planmeca system (Planmeca ProMax 3D Classic, Helsinki, Finland); field of view size 5 x 5 cm; voxel size 0.15 mm and voltage 70-90 kV, with an exposure time of 15 seconds. The software used was Planmeca Romexis Viewer 4.5.2, and the analyses were performed directly on the computer monitor screen with a resolution of 1280 x 1024 pixels. All measurements were performed by one calibrated examiner (AB). Intra-examiner reproducibility was assessed using randomly selected CBCT scans of 17 teeth, measuring the marginal level of the buccal bone with a difference of four weeks. The calculated mean variability between the repeated measurements showed good agreement intraclass correlation coefficient [ICC] = 0.0994), with a coefficient of variation (CV) of 3.6%. These results evidenced the reproducibility of the measurements.

The following bone dimensions were measured from the CBCT scans preoperatively (T0) and one year after surgery (T1) (Fig. 2):

7. Distance from the cementoenamel junction (CEJ) to the buccal bone crest (CEJ-BBC). The difference in this parameter between the two timepoints (T0 and T1) yielded the marginal bone loss of the tooth (MLB).

8. Height from the buccal cortical bone crest to the start of the lesion and/or end of the apex if the lesion has healed after one year (BCL). The possible presence of buccal cortical fenestration was assessed, and its size was measured.

9. Apical depth to the buccal cortical bone (AD). This was defined as the distance from the apex at its middle portion to the buccal cortical bone.

10. Buccal cortical bone thickness (BCT). This was defined as the distance measured perpendicular to the axis of the tooth at 1 mm (BCT1), 3 mm (BCT3) and 5 mm (BCT5) from the buccal bone crest (BBC).

The volume of the lesions was measured from the cross-sectional area of the periapical lesion in the axial plane of the CBCT scan. The lesion was manually outlined using the tool of the program and the area was automatically calculated. The program automatically transforms the number of voxels measured into mm3. In the follow-up images obtained after one year, those in which the periodontal width did not exceed twice the space were recorded as 0 mm3 defects (healed case).

- Outcome measures

Baseline and one-year data were compared to calculate any changes in the assessed clinical (PPD, GM, BOP and CAL) and tomographic parameters (CEJ-BBC, BCL, AD and BCT), using the following formula: change = (preoperative value) - (one-year value).

The correlation coefficients between the clinical and radiological parameters were calculated.

Healing after 12 months of follow-up was judged by one researcher. Based on the clinical and tomographic Modified Penn 3D Criteria, outcomes were classified as success or failure, as follows (11): success = absence of clinical signs and symptoms and radiographic evidence of complete or limited healing, and failure = presence of any clinical signs or symptoms or radiographic evidence of uncertain or unsatisfactory healing.

- Statistical analysis

The statistical analysis described parameters and differences in terms of means, standard deviations and medians. Due to the lack of normal distributions, nonparametric tests were used. The Wilcoxon test was conducted to assess changes in the periodontal and radiographic parameters before and after surgery. The Mann-Whitney U-test in turn was used to analyze differences in distributions across gender or type of tooth. Spearman coefficients were obtained for estimating non-linear correlation between CAL and radiological MLB. The significance level was set at 5% (*p*=0.05). All analyses were performed using the SPSS version 19.0 statistical package (IBM Corp., Somers, NY, USA).

## Results

A total of 41 patients and 41 treated teeth were initially enrolled in the study. At one year of follow-up, 36 cases remained for analysis. Reasons for dropouts (*n*=5) were: three patients lacked tomographic control at 12 months (2 patients could not be located, and one patient was pregnant and CBCT could not be performed); one patient suffered root fracture 6 months after surgery; and one patient underwent filling of the bone cavity with bone substitute.

The 36 patients included in the final analysis (19 woman and 17 men) had a mean age of 43.1 ± 14.8 years; there were 36 anterior maxillary teeth (18 central incisors, 13 lateral incisors and 5 canines).

- Clinical assessment

Before surgery, 8 patients were asymptomatic, 10 had pain, 7 presented swelling, and 11 suffered both pain and swelling. There were no alterations of the soft tissue in 21 cases, while 7 patients had swelling and 8 presented a fistula. One year after surgery there were no soft tissue alterations, while two patients reported altered sensation not classified as pain, and which did not affect their daily activity.

The changes in PPD at four sites were only minimal over the observation period of one year, with a mean midbuccal measurement reduction between the pre- and postoperative timepoints of 0.14 ± 0.76 mm. One year after surgery, BOP was found to improve, with no bleeding in 74.19% of the cases, isolated bleeding spots in 22.58%, and blood lines in only 3.22%. Gingival recession and attachment loss were 0.19 mm and 0.28 mm, respectively (Table 1).

No aesthetic changes were observed, as there was no soft tissue scarring after surgery, and gingival recession at one year was less than (0.19 ± 0.33 mm). None of the patients complained about aesthetic problems.

- Tomographic assessment

The descriptive results of the measurements are shown in Table 2. After one year of follow-up, the distance from the cementoenamel junction to the buccal bone crest (CEJ-BBC) increased significantly with respect to the preoperative value, from 2.49 mm to 2.79 mm; the difference between these two timepoints yielded the marginal bone loss (MLB) of the tooth (0.25 mm).

In the preoperative CBCT scan, the height from the buccal cortical bone crest to the start of the lesion (BCL) was 7.44 ± 2.92 mm. The buccal cortical bone was seen to be intact in 19 cases, and 17 teeth presented fenestration, measuring 3.92 ± 1.99 mm on average (range 0.60-6.60); this coincided with the number of fenestrations clinically identified on the day of surgery. Following appraisal of the one-year postoperative volumes, 5 patients had buccal cortical bone that remained unhealed, and two patients had the root apex outside the biological boundary of the buccal cortical bone contour.

In terms of the different types of teeth, the preoperative mean thickness of the buccal cortical bone at 1 mm to the bone crest (BCT1) for the central incisors, lateral incisors and canines was 0.84 mm, 0.80 mm and 1.15 mm, respectively, versus 0.61 mm, 0.75 mm and 0.95 mm after one year of follow-up. In turn, the preoperative mean thickness of the buccal cortical bone at 3 mm to the bone crest (BCT3) was 1.50 mm, 0.65 mm and 1.19 mm, respectively, versus 0.65 mm, 1.10 mm and 1.16 mm after one year of follow-up. Lastly, the preoperative mean thickness of the buccal cortical bone at 5 mm to the bone crest (BCT5) was 0.80 mm, 0.60 mm and 1.16 mm, respectively, versus 0.67 mm, 1.05 mm and 1.09 mm after one year of follow-up. One year after surgery, the thickness of the buccal cortical bone was seen to have decreased at all the measurement points, with the largest decrease corresponding to BCT3.

The distance from the apex to the buccal cortical bone (apical depth [AD]) showed a decrease of 28.5% one year after surgery (0.59 ± 1.16 mm).

- Outcome measures

No significant increase in CEJ-BBC was observed between the preoperative and one-year postoperative measurements for PPD or CAL (Table 2). The correlation between CAL and the marginal buccal bone was weak before surgery (r=0.36; *p*=0.072) and weak to moderate after one year of follow-up (r=0.48; *p*=0.019).

The initial mean volume of the lesions of the anterior maxillary teeth was 457 mm³, and this value decreased to 28.4 mm³ one year after surgery, with a significant mean reduction of 428.6 mm³ (93.8%). The successful healing rate was 94.4%.

## Discussion

The present study evaluated the changes in periodontal, tomographic and aesthetic parameters one year after apical surgery in anterior maxillary teeth with chronic apical periodontitis. A number of studies (5,8) have reported that the level of the gingival margin varies according to the type of incision used, with submarginal incisions being associated with a lesser risk of midbuccal gingival recession (3). For this reason, and to ensure more homogeneous results, we performed submarginal incision in all cases. Kreisler *et al*. (1) reported a grater incidence of scarring with submarginal incisions, though in the present study no scars were observed after surgery. The explanation for this could be attributed to differences in operator experience, knowledge and skill; caution is therefore required when interpreting the results. In our study, none of the patients had aesthetic complaints, and none of the teeth presented major clinical aesthetic changes (recession < 1 mm and no wound scarring).

Only one previous clinical study was found comparing clinical periodontal parameters with the three-dimensional (3D) radiographic findings in apical surgery (12). The authors reported a change in periodontal clinical attachment level at the midbuccal site of 0.06 mm, and a change in gingival margin 0.14 mm. These same authors (7) showed patients in the submarginal incision group to have a change in gingival margin of 0.05 ± 0.61 mm at one year after surgery. Verardi (13) in turn found that the changes in gingival margin at one year remained without change 5 years after apical surgery. Gingival recession associated with the intrasulcular incision was 0.47 mm, versus 0.31 mm for the base of the papilla, and 0.12 mm at submarginal level. In our study, submarginal incision resulted in CAL 0.28 mm and similar gingival recession (0.19 mm) after one year of follow-up.

In the meta-analysis published by Rojo *et al*. (14), the mean CEJ-BBC was 2-2.5 mm for all the analyzed teeth, and the mean thickness of the buccal cortical bone was ≤ 1 mm for the upper canines and incisors (0.75-1.05 mm). These data are consistent with our own findings. The marginal bone loss in the present study was greater than that reported by von Arx *et al*. (12), but without clinical relevance (0.25 mm versus 0.15 mm, respectively).

Kopacz *et al*. (15) identified fenestrations in 91% of the CBCT scans, with a mean size of 19.6 ± 33.2 mm2. In the present study we identified 17 fenestrations on the day of surgery, in coincidence with the number detected by CBCT. Based on 3D radiographic assessments, the BCT in the anterior teeth ranged between 0.5 and 1.50 mm (14,16), with the majority of cases being associated with a wall thickness of ≤ 1 mm (63% (6) to 69% (17)). Ramanauskaite *et al*. (18) found 87% of the teeth in the anterior maxilla to have a facial alveolar bone wall thickness of ≤ 1 mm, with a mean value of 0.8 mm. Regarding the different categories of tooth sites, Rojo-Sanchis *et al*. (14) found the mean buccal bone thickness in the case of the central incisor to be 0.72 mm, versus 0.81 mm for the lateral incisor and 0.83 mm for the canine region. Ghassemian *et al*. (19) in turn measured buccal bone thickness on 66 CBCT scans at 2 mm apical to the crest, and recorded values of 0.39 mm for the canines, 1.28 mm for the lateral incisors, and 1.22 mm for the central incisors. In their study, the BCT increased from the bone crest to the apical area, whereas in the present study the thickest facial bone was detected at 2 mm below the crest and then at 5 mm apical to the crest and at the crest, respectively. We recorded a preoperative mean BCT of 0.87, 1.37 and 0.88 mm as measured 1, 3 and 5 mm from the BBC, respectively.

A study of tissue healing, based on radiographic changes, showed a direct relationship between the size of the lesion and the healing time. A lesion of less than 5 mm will take an average of 6.4 months to repair, versus 7.25 months for a lesion measuring 6-10 mm, and 11 months for lesions over 10 mm in size (20). For this reason, our study established a control at 12 months after surgery. Our mean lesion volume before apical surgery was 457 mm³ versus 28.4 mm³ at one year of follow-up, and the successful healing rate was 94.4%. Ramis-Alario *et al*. (21), in a series of 57 teeth, recorded a mean preoperative volume of 147.7 mm3, with a postoperative volume of 15.5 mm3, corresponding to a volume healing rate of 6.2 mm3 per month (79.1% decrease in total volume). Their success rate after two years of follow-up was 93%, while Kim *et al*. (11) reported a success rate of 88.7%.

In relation to the limitations of the present study, mention must be made of the need to include greater sample sizes in future studies, with the involvement of different operators.

## Conclusions

One year after apical surgery of anterior maxillary teeth with submarginal incision, only minimal clinical periodontal and tomographic changes are observed, with no clinical relevance. The mean lesion volume decreased 93.8%, and the success rate was 94.4%.

## Figures and Tables

**Table 1:** Periodontal parameters preoperatively and at one year of follow-up (n=36).

PERIODONTAL PARAMETERSN=36		PREOPERATIVEBaseline data(mean ± SD, mm)	ONE-YEAR DATA(mean ± SD, mm)	CHANGES(mean ± SD, mm; p-value for comparison preoperative versus one-year data)
PPD	MB	2.30 ± 0.76 (median 2.0)	2.44 ± 0.70 (median 2.0)	0.12 ± 0.39 (p=0.130)
	MD	2.30 ± 0.91 (median 2.0)	2.18 ± 0.95 (median 2.0)	-0.14 ± 0.76 (p=0.473)
	DB	2.37 ± 0.63 (median 2.0)	2.58 ± 0.59 (median 3.0)	0.18 ± 0.45 (p=0.083)
	P	2.33 ± 0.83 (median 2.0)	2.40 ± 0.82 (median 2.0)	0.08 ± 0.28 (p=0.157)
GM		-0.5 ± 0.83	-0.69 ± 0.87	-0.19 ± 0.33 (p=0.003**)
BOP		Absence 57.1%Isolated spots 35.7%Blood line 7.1%	Absence 74.19%Isolated spots 22.58%Blood line 3.22%	Absence 6.45%Isolated spots 3.22%Blood line3.18%
CAL=PPD MD - GM		2.50 ± 1.19 (median 2.0)	2.78 ± 1.32 (median 3.0)	-0.28 ± 0.85 (median 0.0) (p=0.14*)

**Table 2:** Tomographic measurements before and one year after apical surgery (n=36).

CASES (N=36)		PREOPERATIVEBaseline data(mean ± SD, mm)	ONE-YEAR DATA(mean ± SD, mm)	CHANGES(mean ± SD, mm; p-value for comparison preoperative versus one-year data)
CBCT PARAMETERS	CEJ-BBC	2.49 ± 0.96 (median 2.24)	2.79 ± 0.92 (median 2.70)	-0.25 ± 0.68 (median 0.12)(p=0.029*)
	BCL	7.44 ± 2.99 (median 7.00)	6.36 ± 2.81 (median 5.91)	-1.04 ± 3.48 (median-1.35)(p=0.104)
	BCT 1 mm	0.87 ± 0.44 (median 0.80)	0.68 ± 0.51 (median 0.75)	-0.19 ± 0.55 (median 0.09)(p=0.011*)
	BCT 3 mm	1.37 ± 2.00 (median 0.90)	0.81 ± 0.66 (median 0.75)	-0.58 ± 2.03 (median -0.1) (p=0.040*)
	BCT 5 mm	0.88 ± 0.52 (median 0.75)	0.72 ± 0.82 (median 0.75)	-0.22 ± 0.76 (median-0.06) (p=0.348)
	AD	2.87 ± 1.11 (median 2.85)	2.12 ± 0.99 (median 1.89)	-0.59 ± 1.16 (median -0.43)(p=0.001**)
SPEARMAN CORRELATION COEFFICIENT (CLINICAL/TOMOGRAPHIC)	PPD with CEJ-BBC	r = 0.36 (p=0.072)	r=0.48 (p=0.019*)	r=0.36 (p=0.091)
	CAL with CEJ-BBC	r = 0.49 (p=0.011*)	r=0.55 (p=0.006**)	r=0.44 (p=0.036*)
	LESION VOLUME	457.0 ± 791.9 mm³(median 134.5)	28.4 ± 69.1mm³(median 0.0)	-428.6 ± 788.0 mm³(median -134.5)
	SUCCESS RATE	-	-	94.4% (p<0. 001***)

Correlation of clinical and radiographic parameters Spearman correlation coefficient, r (p-value). CEJ-BBC (distance from the cementoenamel junction to the buccal bone crest), BCL (height from the buccal cortical bone crest to the start of the lesion), BCT (buccal cortical bone at 1, 3 and 5 mm), AD (apical depth to the buccal cortical bone), PPD (pocket probing depth), CAL (clinical attachment level). (*p<0.05, ** p<0.01, *** p<0.001).
